# miR‐181a/b control the assembly of visual circuitry by regulating retinal axon specification and growth

**DOI:** 10.1002/dneu.22282

**Published:** 2015-06-11

**Authors:** Sabrina Carrella, Ylenia D'Agostino, Sara Barbato, Sabina P. Huber‐Reggi, Francesco G. Salierno, Anna Manfredi, Stephan C.F. Neuhauss, Sandro Banfi, Ivan Conte

**Affiliations:** ^1^ Telethon Institute of Genetics and MedicineVia Campi Flegrei 34, Pozzuoli (Naples)80078Italy; ^2^ Institute of Molecular Life Sciences, Division of Neurobiology, University of ZurichWinterthurerstrasse 190CH‐8057ZurichSwitzerland; ^3^ Medical Genetics, Department of BiochemistryBiophysics and General Pathology, Second University of Naplesvia Luigi De Crecchio 780138NaplesItaly; ^4^ Present address: Stazione Zoologica Anton DohrnVilla Comunale 8012180131NaplesItaly

**Keywords:** miR‐181a, miR‐181b, axon specification, retina, MAPK signaling

## Abstract

Connectivity and function of neuronal circuitry require the correct specification and growth of axons and dendrites. Here, we identify the microRNAs miR‐181a and miR‐181b as key regulators of retinal axon specification and growth. Loss of miR‐181a/b in medaka fish (*Oryzias latipes*) failed to consolidate amacrine cell processes into axons and delayed the growth of retinal ganglion cell (RGC) axons. These alterations were accompanied by defects in visual connectivity and function. We demonstrated that miR‐181a/b exert these actions through negative modulation of MAPK/ERK signaling that in turn leads to RhoA reduction and proper neuritogenesis in both amacrine cells and RGCs via local cytoskeletal rearrangement. Our results identify a new pathway for axon specification and growth unraveling a crucial role of miR‐181a/b in the proper establishment of visual system connectivity and function. © 2015 Wiley Periodicals, Inc. Develop Neurobiol 75: 1252–1267, 2015

## INTRODUCTION

An important feature of the central nervous system (CNS) is the organization of neural connections into discrete layers, or laminae, that convey different types of information. This is well exemplified in the retina, in which, neurons are organized into laminae with each neuronal class adopting specific patterns of connectivity in the appropriate sublamina. The specification and growth of retinal axons and dendrites are essential steps to ensure the correct neuronal circuitry and layer formation. This highly polarized process requires rapid and local changes in cytoskeletal organization and plasma‐membrane components. External guidance cues control the neurite growth‐cone extension and retraction processes through intracellular protein phosphorylation, local protein degradation, and synthesis of cytoskeletal regulators. These events are coordinated by several signaling pathways (e.g., Netrin/DCC, Ephrin/EPH, Sonic hedgehog, Wingless (WNT), Transforming Growth Factor/Bone Morphogenetic Protein (TGF‐β/BMP), Mitogen Activated Protein Kinase (MAPK), etc.) (Bovolenta, 2005; Sanchez‐Camacho and Bovolenta, 2009; Jung et al., 2012), that are highly interconnected and translate the external information into well‐established dose‐dependent responses. The integration among these pathways is controlled by many feedback and feed‐forward loops, which render their functionality more similar to a network rather than to a linear cascade. However, the molecular basis and the identity of the modulators of the above pathways are still largely unknown.

MicroRNAs (miRNAs) represent attractive candidates as effectors of feed‐forward and feed‐back circuits in the regulation of signaling pathways in retinal differentiation and layering. They are a class of 20‐ to 25‐nucleotide small noncoding RNA molecules that have basic roles in post‐transcriptional regulation of gene expression. Indeed, miRNAs are emerging as nodes of signaling networks that ensure fundamental cellular programs, such as cell proliferation and differentiation, and programmed cell death (Inui et al., 2012). Increasing evidence suggests the involvement of miRNAs in the control of neuron differentiation (Fineberg et al., 2009; Liu and Zhao, 2009; Li and Jin, 2010), and neuronal axon pathfinding (Baudet et al., 2012), extension and branching (Dajas‐Bailador et al., 2012; Zhang et al., 2013).

Among the miRNAs with high CNS expression, two members of the miR‐181 family, miR‐181a, and miR‐181b, show intriguing spatio‐temporal expression domains, with high levels in the retina, particularly in retinal ganglion cells (RGCs) and the inner cell layers, and in brain areas associated with visual function (Ryan et al., 2006; Kapsimali et al., 2007; Karali et al., 2007). This suggests that they could have roles in defining the connectivity of the visual system. By analyzing the functional consequences of gain‐of‐function and loss‐of‐function approaches in the medaka fish [*Oryzias latipes* (ol)], we show that miR‐181a and miR‐181b govern axon specification and growth by fine regulating MAPK/ERK signaling and are key players in the organization of neural connections in the retina.

## RESULTS

### MiR‐181a and miR‐181b are Essential for Correct Inner Plexiform Layer Formation in the Retina

In the medaka fish genome, the miR‐181a and miR‐181b are organized in at least four different cluster that are localized to different genomic loci, that is, on chromosome 4, chromosome 9, chromosome 17, and on the Ultracontig105 (http://genome.ucsc.edu/). These two miRNA members differ only by three nucleotides located outside a completely identical seed sequence (Supporting Information Fig. S1a). We observed that miR‐181a and miR‐181b show overlapping expression patterns during medaka embryo development. Both miR‐181a and miR‐181b localized to differentiating amacrine and ganglion cells of the neural retina from stage (St) 30, that is, when the retinal Inner Plexiform Layer (IPL) forms [Supporting Information Fig. S1(b–e')], and to different regions of the CNS, including the pretectal and tectal areas, which are visual areas in vertebrates [Supporting Information Fig. S1(f–g')]. This expression pattern suggested that both miR‐181a and miR‐181b contribute to establishment of the correct neuronal circuitry in the visual system.

To investigate this possibility further, we interfered with miR‐181a and miR‐181b activity using a morpholino (MO)‐based knock‐down approach (Conte et al., 2010a, 2010b). To this end, two specific MO oligonucleotides (MO‐miR‐181a and MO‐miR‐181b, Supporting Information see Table S1) were designed to sterically block, respectively, all the miR‐181a and miR‐181b mature sequence, derived from the different miR‐181‐family clusters. The specificity of the MO used was verified with all of the recommended controls (Robu et al., 2007; Eisen and Smith, 2008) (see Supporting Text S1, Supporting Information Fig. S2 and Supporting Information Table S1). The MO‐miR‐181a and Mo‐miR‐181b were injected into fertilized one‐cell medaka embryos either alone or in combination.

**Figure 1:**
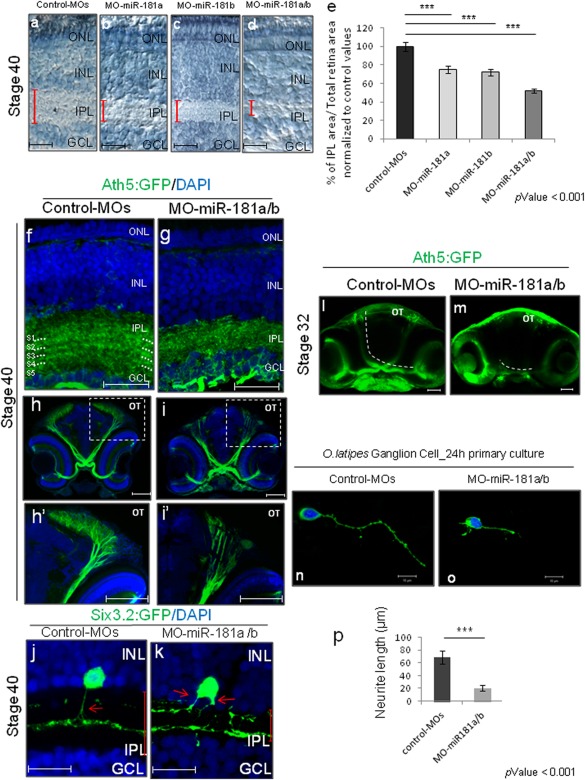
miR‐181a and miR‐181b are essential for correct neuronal circuitry in the visual system. **(a–d)** Retinal frontal sections of St38 control (a), miR‐181a morphant (b), miR‐181b morphant (c), and miR‐181a/b morphant (d) embryos processed for Richardson‐Romeis staining. Red bars, IPL thickness. Scale bars: 20 µm. **(e)** Quantitative analysis of IPL thickness, defined as the mean ratio in the central retina between the IPL and the total retinal area normalized to IPL area on Total retina area ratio of Control‐MOs (*n* = 50). Data are means ±s.e.m. ***, *p* < 0.001 (*t*‐tests). Note that the miR181a/b morphant embryos show 48% decrease in IPL thickness when compared with control MOs. **(f–i')** Representative frontal sections from St38 control MO (f–f', h–h') and miR‐181a/b morphant (g–g', i–i') Ath5:eGFP transgenic medaka embryos. Cell nuclei are stained with DAPI (blue); GFP (green) stains the RGC soma and neurites. f', g', h', i': higher magnifications of boxed areas in f, g, h, and i, respectively. **(f–g')** The IPL sublaminae division in the miR181a/b morphant retina was altered when compared with the control retina. Dotted lines, sublaminae S1–S5. Scale bars: 20 µm. **(h–i')** Axonal processes from eGFP‐labeled RGCs in Ath5:eGFP control‐MO transgenic embryos extended medially toward the optic chiasm, and ascended to the optic tectum (OT). In St38 miR‐181a/b–depleted transgenic embryos, the optic nerve correctly crossed the optic chiasm and reached the brain, but showed marked reduction of axonal branching in the OT, when compared with control‐MOs. Scale bars: 100 µm. **(j–k)** Representative images of amacrine cells from St38 retinal sections of control‐MOs (j), and of miR‐181a/b morphant Six3:eGFP transgenic embryos (k). Cell nuclei are stained with DAPI (blue). GFP (green) stains amacrine cell soma and neurites. In control‐MO embryos (j), eGFP‐expressing amacrine cells send a single axon‐like structure (red arrow) into the IPL. In miR‐181a/b morphant Six3:eGFP transgenic embryos (k), GFP‐labeled amacrine cells show altered morphology, with multiple neurites (red arrows) that extend from the cell soma towards the IPL. Red bars in j–k, IPL thickness. Scale bars: 20 µm. ONL, outer nuclear layer; INL, inner nuclear layer; GCL, ganglion cell layer. **(l–m)** Representative 2‐D reconstruction of confocal images of St32 control‐MO (l) and miR‐181a/b morphant (m) Ath5:eGFP transgenic whole‐heads. RGC axonal processes of miR‐181a/b morphants correctly cross the optic chiasm, but fail to reach the optic tectum (OT) (m). Dotted white lines, route of optic nerve in control and miR‐181a/b morphants. Scale bars: 50 µm. **(n–o)** Representative images of primary RGC cultures from St30 control (n) and miR‐181a/b morphant (o) transgenic embryos. After 24 h, the axon length of morphant RGCs was shorter than in the control. Scale bars: 10 µm. **(p)** Quantification of RGC axonal length. Data are means +s.e.m (*n* = 100) from three independent cell culture experiments (see online Methods). ***p < 0.001 (*t*‐test). [Color figure can be viewed in the online issue, which is available at wileyonlinelibrary.com.]

At early stages of development, the eyes of all of these morphant medaka embryos were morphologically indistinguishable from those of mismatched (mm)‐MO‐miR‐181a/b‐injected embryos (hereinafter indicated as control MOs). However, from St32 onward, a reduction in the thickness of the retinal IPL was detected in the morphant embryos in which either miR‐181a or miR‐181b had been knocked down. This eye phenotype culminated in an evident reduction in the IPL thickness at St40 [53% ± 5% and 41% ± 5% of MO‐miR‐181a– and MO‐miR‐181b–injected embryos, respectively; *n* = 300 for each; Fig. 1(a–c, e)]. The same phenotype was observed in the double‐morphant embryos (MO‐miR‐181a/b), with similar onset and progression, but with considerably higher penetrance [92% ± 3% of inspected embryos; *n* = 900; Fig. 1(d,e)] and considerable reduction of the IPL thickness, when compared to the single miR‐181a and miR‐181b depletions [Fig. 1(a–e)]. These data suggest functional redundancy of the two miRNAs, which share the same “seed region,” and are therefore predicted to target the same set of genes (Gennarino et al., 2012). Thus, to avoid that this redundancy blurred the full understanding of their *in vivo* roles, as observed for other miRNA families (Wang et al., 2008; Wei et al., 2014), we focused on the characterization of the double miR‐181a/b morphant embryos.

### Inactivation of miR‐181a/b Alters Amacrine and RGC Neuritogenesis

**Figure 2:**
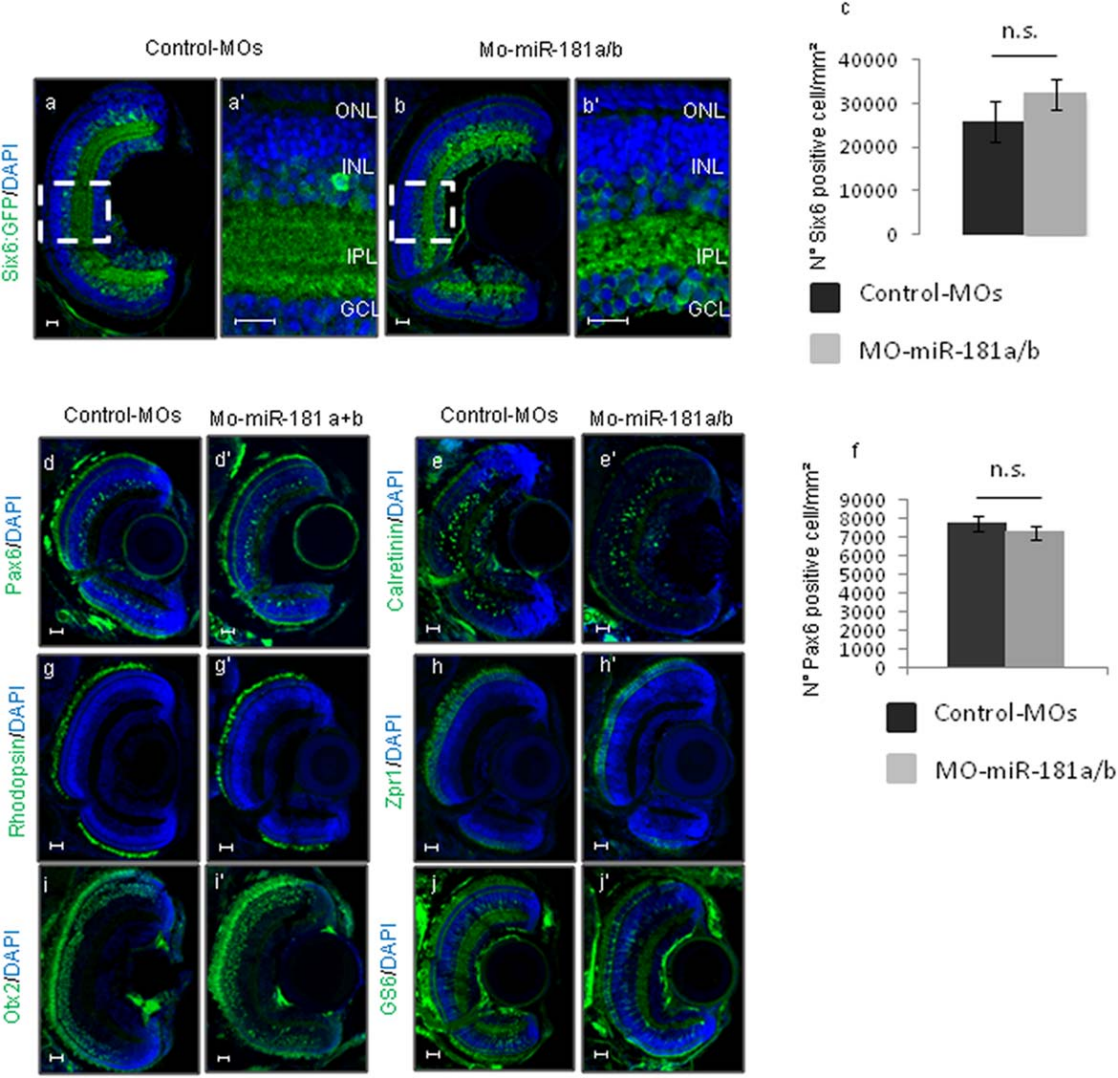
MiR‐181a/b down‐regulation does not alter retinal cell differentiation. **(a–c)** Representative images control‐MOs and MO‐miR‐181a/b retinas of Six6:GFP transgenic embryos (a–b'), highlighting the structural disorganization of the IPL in the MO‐miR‐181a/b–injected retinas. **(a'–b')** Magnifications of the dotted boxes in (a–b), respectively. ONL, outer nuclear layer; INL, inner nuclear layer; IPL, inner plexiform layer; GCL, ganglion cell layer. Scale bars: 20 µm. **(c)** Quantification of Six6‐positive amacrine cells. Data are means ± standard deviation (*n* = 20, for each). **(d–e')** Representative immunofluorescence images of control‐MOs and MO‐miR‐181a/b retinas at St38, stained with different retinal markers (green) and DAPI (blue). No differences are visible in the sections stained with the amacrine markers Pax6 (d, d') and Calretinin (e, e'). Scale bars: 20 µm. (f) Quantification of Pax6‐positive amacrine cells. Data are means ± standard deviation (*n* = 20, for each). **(g–j')** Representative immunofluorescence images of control‐MOs and MO‐miR‐181a/b retinas at St38, stained with different retinal markers (green) and DAPI (blue). No differences are visible in the sections stained with the photoreceptor markers Rhodopsin (g, g') and Zpr1 (h, h'), with the bipolar marker Otx2 (i, i') and the Muller glial cell marker GS6 (j, j'). Scale bars: 20 µm. [Color figure can be viewed in the online issue, which is available at wileyonlinelibrary.com.]

The IPL of the vertebrate retina is established by the temporally organized growth of RGC dendrites, followed by that of the processes of amacrine cells and of bipolar cell axons, the synapses of which are arranged into discrete layers. At St40, the IPL of the miR‐181a/b morphants was abnormally thin, and also showed defects in sublaminae organization, as revealed by the characterization of MO‐miR‐181a/b‐injected Six6:eGFP (enhanced Green Fluorescent Protein) and Ath5:eGFP transgenic medaka lines, in which the amacrine cell and RGC processes, respectively, were GFP labeled [Fig. 1(f,g), and Fig. 2(a–b')]. We also observed abnormal defasciculation of the optic nerve in the pretectal area, and reduced and irregular projections of the RGC axons to the optic tectum of MO‐miR‐181a/b‐injected Ath5:eGFP transgenic fish [66% ± 5% of dissected embryos; *n* = 250; Fig. 1(h–i')]. These data suggest that miR‐181a/b are essential to ensure the appropriate connectivity between RGCs and their target cells in the optic tectum. Of note, we observed no differences in the numbers of differentiated amacrine cells in morphant retinas, as determined by analysis of the Six6:eGFP transgenic line at St40 [Fig. 2(a–c)] and by Pax6 and Calretinin immunofluorescence staining [Fig. 2(d–f)], which excluded the possibility that the IPL defect is due to decreased numbers of amacrine cells. Similarly, there were no apparent differences between morphants and control MO‐injected embryos in the differentiation of rod and cone photoreceptors, bipolar cells, RGCs, and Muller glial cells, which indicated that the miR‐181a/b knock‐down does not affect overall retinal cell differentiation [Fig. 2(g–j')].

Correct specification and growth of axons and dendrites from different neurons are a prerequisite for correct neural circuit assembly (Robles and Baier, 2012). Therefore, we sought to determine whether the miR‐181a/b morphant phenotype is related to abnormal neuritogenesis and axon growth of amacrine cells and RGCs. Consistent with this hypothesis, in Six3:eGFP transgenic fish, the eGFP‐positive amacrine cells, which normally generate a single axon‐like process that extends within the IPL, showed multiple but normally oriented processes after injection of MO‐miR‐181a/b at St38 [42% ± 3% of amacrine cells in Six3:eGFP dissected embryos; *n* = 200; Fig. 1(j–k)]. To examine the defects seen in the morphology of amacrine cell processes in more detail, we established primary cultures of amacrine cells from St32 Six6:eGFP medaka transgenic embryos. Within 24 h, the vast majority of eGFP‐positive amacrine cells from control MOs developed a single axon‐like process (94.8% ± 3%; *n* = 100), as shown by p‐Tau1 staining [Supporting Information Fig. S3(a–d)]. In contrast, 45% ± 5% of the amacrine cells from miR‐181a/b morphants (*n* = 100) failed to form a p‐Tau1‐positive axon‐like process [Supporting Information Fig. S3(a'–d')]. Overall, these data show that knock‐down of miR‐181a/b causes structural abnormalities in the developing IPL, and that miR‐181a/b activity is required for neuritogenesis and specification of axon‐like structures in amacrine cells.

We next investigated the process of RGC axon elongation in miR‐181a/b morphants, by taking advantage of the Ath5:eGFP transgenic cell line. At St32, there was reduced axon growth in morphant RGCs, in comparison with the control MO treatment [66% ± 5% of MO‐miR‐181a/b–inspected Ath5:eGFP embryos; *n* = 250; Fig. 1(l–m)]. This defect was not a consequence of incorrect timing of onset and progression of RGC differentiation [Supporting Information Fig. S4(a–d)], which supports a direct role of miR‐181a/b in RGC axon growth. Indeed, the axons of the miR‐181a/b morphant RGCs were significantly shorter than those of the control MOs in 24‐h RGC primary cultures from St30 Ath5:eGFP transgenic embryos [Fig. 1(n–p)]. These findings indicate that miR‐181a/b control RGC axon elongation during retino‐tectal pathfinding.

Overall, we can conclude that miR‐181a/b are necessary for correct neuritogenesis in both amacrine cells and RGCs, and they thus contribute to the establishment of the appropriate connectivity of the visual system.

### Knock‐Down of miR‐181a and miR‐181b Alters Visual Functionality

**Figure 3:**
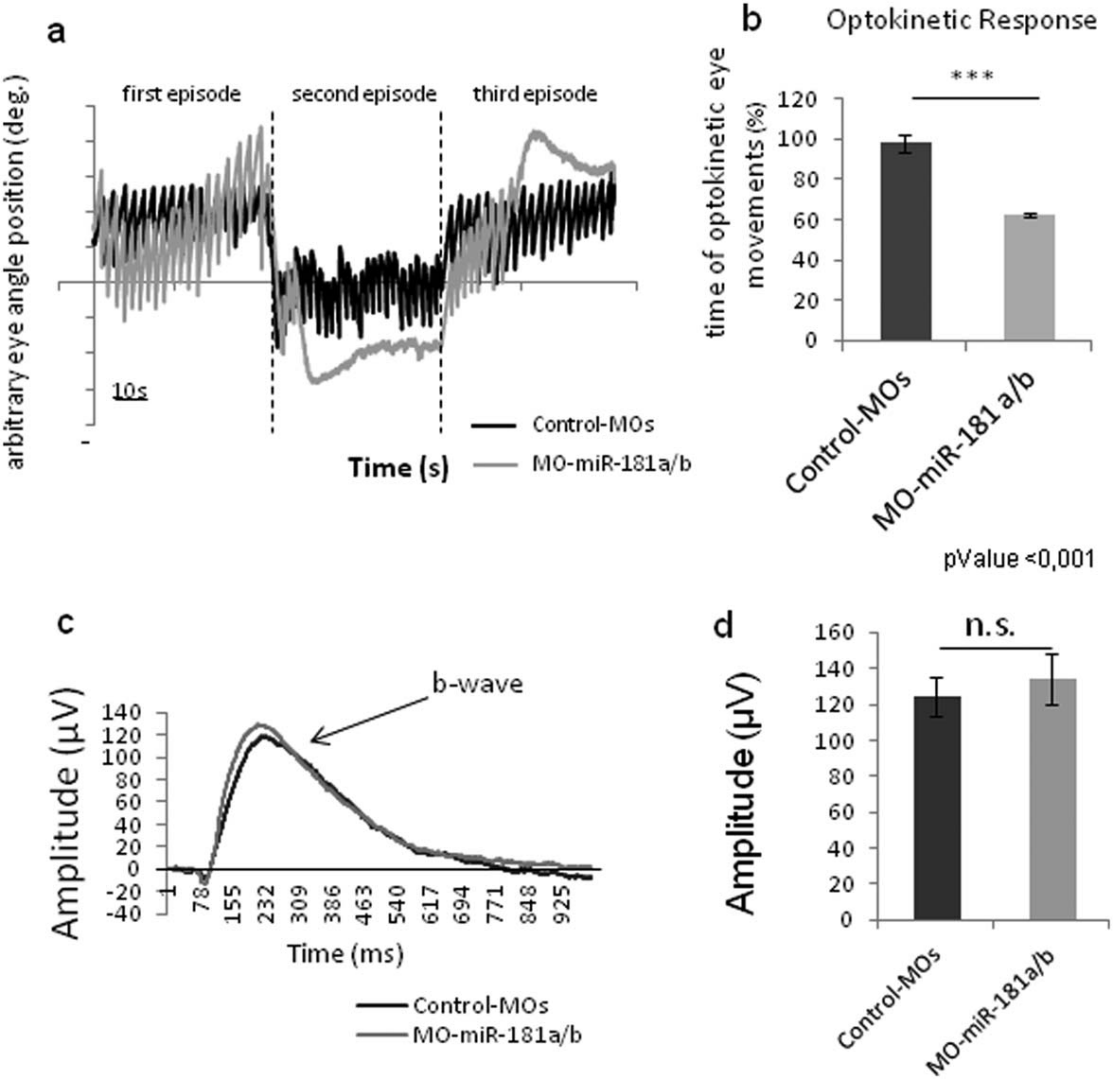
Knock‐down of miR‐181a and miR‐181b alters visual functionality but not the electroretinogram. **(a)** Representative trace of the eye angle position of control‐MOs (black line) and miR‐181a/b morphant (gray line) medaka larvae during optokinetic stimulation. After the first episode of 60 s, the stimulus direction was reversed, and returned to the original direction in the third episode. Morphant larvae fail to maintain optokinetic eye movements over the entire stimulation period. **(b)** The impairment of saccadic movement has been quantified as percentage of time with optokinetic eye movement during the time of stimulation. Data are means ±SEM (*n* = 20, for each). MiR‐181a/b morphant larvae show significant loss of saccadic movement. ***, *p* < 0.001 (unpaired *t*‐test) (see Supplementary Movies and Methods). **(c)** Averaged ERG traces of dark‐adapted control‐MOs (black line) and MO‐miR‐181a/b (gray line) larvae for a stimulus of 665 lux. The ERG shows the small a‐wave, which reflects photoreceptor activation, and the b‐wave, which reflects the activity of ON bipolar cells (as indicated). The ERG was not altered in the MO‐miR‐181a/b larvae. **(d)** Quantification of b‐wave amplitude of control and MO‐miR‐181a/b larvae, as shown in (c). Data are means ± SEM (*n* ≥20). The mean b‐wave amplitude did not significantly differ between MO‐miR‐181a/b and the control larvae. n.s., *p* = 0.59 (unpaired *t*‐test). [Color figure can be viewed in the online issue, which is available at wileyonlinelibrary.com.]

To investigate whether the neuritogenesis defects in miR‐181a/b morphant embryos are also reflected in alterations to visual function, we quantified the optokinetic response (OKR), which is a compensatory ocular motor reflex in response to a moving whole‐field visual environment. Eye movements during OKR are characterized by cycles of slow tracking movements in the direction of the visual environment and resetting saccadic movements in the opposite direction. Correct OKR performance requires a well‐assembled visual circuit, which includes a correctly formed IPL and retino‐tectal connectivity (Neuhauss et al., 1999; Huang et al., 2006; Schoonheim et al., 2010; Huber‐Reggi et al., 2012). OKR analysis at 10 days postfertilization showed that 90% ± 5% of miR‐181a/b morphant larvae (*n* = 20) failed to maintain saccadic resetting movements and to alternate correctly from the slow to the quick phase of eye movement cycles, compared to the control MOs [Supporting Information Movie S1–S2, Fig. 3(a–b)]. To quantify the electrical activity of the outer retina, we recorded electroretinograms, which did not show any differences between the morphant and control MO larvae [Fig. 3(c–d)]. This supports the observed normal development of the photoreceptor and bipolar layers following miR‐181a/b knock‐down. Altogether, these functional data indicate that miR‐181a/b do not affect the functionality of the outer retina, and particularly of cone photoreceptors, although they are necessary for correct visual function through their contribution to the correct inner retinal and retino‐tectal connectivity.

### MiR‐181a/b‐Mediated Regulation of MAPK/ERK Signaling Controls Retina Neuritogenesis

**Figure 4:**
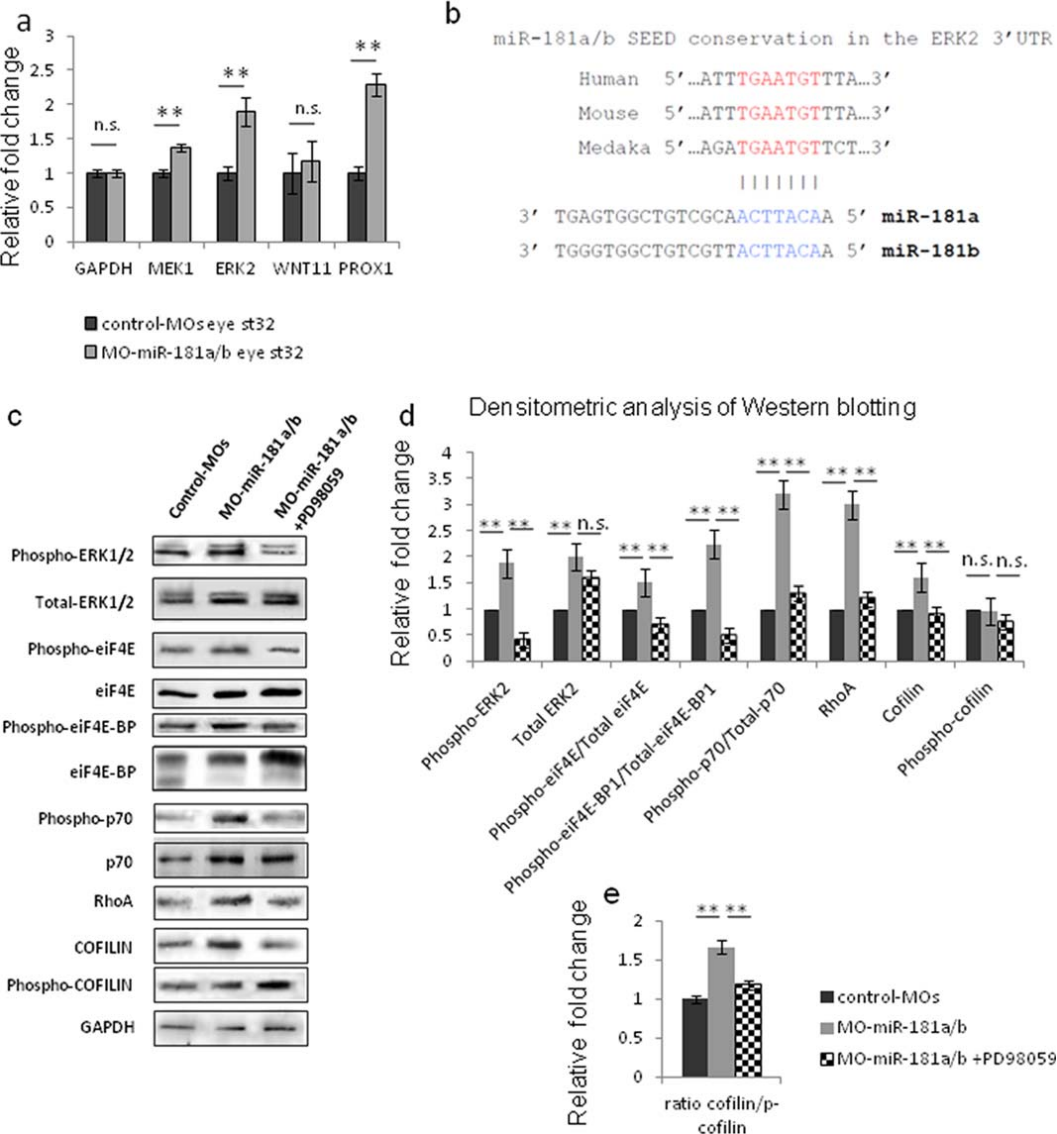
MiR‐181a/b control MAPK‐ERK signaling. **(a)** qRT‐PCR on total RNA from St32 control and miR‐181a/b morphant eyes, for Erk2, Wnt11, and Prox1 transcripts, normalized to GAPDH transcript levels. Mek1 and Erk2 levels were increased in morphant eyes. Prox1 is an already validated miR‐181 target, and represents the positive control, whereas Wnt11 represents the negative control. **(b)** Graphical representation of miR‐181 family seed sequence conservation in human, mouse and medaka genome. **(c–e)** Representative Western blotting (c) and its quantification (d) show increased ERK2 protein levels in St32 miR‐181a/b morphant, compared with control‐MO medaka eyes. Increases in the active phosphorylated forms of ERK2 and its downstream effectors are seen; that is, phospho‐eiF4E, phospho‐eiF4E‐BP, and phospho‐p70/S6K, normalized to the corresponding total form. The final effect of increased MAPK/ERK signaling is up‐regulation of cofilin (ADF) and RhoA protein levels. Administration of PD98059, a MEK1 inhibitor, to miR‐181a/b morphant embryos results in rescue of MAPK/ERK signaling, with the exception of the total ERK2 protein levels. **(e)** The ratio between active dephosphorylated form of cofilin and its inactive phosphorylated form is increased in morphant eyes, indicating increased cofilin activity. Treatment with PD98059 restores this ratio to control‐MO values. Data are means +s.e.m. **p < 0.05 (*t*‐test). [Color figure can be viewed in the online issue, which is available at wileyonlinelibrary.com.]

To dissect out the molecular mechanisms underlying the miR‐181a/b involvement in retinal neuritogenesis, we searched for possible targets that are known to participate in this process. Part of the neuritogenesis defects triggered by miR‐181a/b knock‐down (Fig. 1) were reminiscent of those observed after alteration of MAPK/ERK signaling activation, which causes neurite retraction and RGC growth‐cone collapse in response to repulsive guidance cues (Perron and Bixby, 1999; Campbell and Holt, 2001; Campbell et al., 2001; Campbell and Holt, 2003; Piper et al., 2006). Indeed, quantitative real‐time RT‐PCR (qRT‐PCR) analysis indicated significantly higher levels of both *Erk2* and *Mek1* transcripts in miR‐181a/b morphant eyes, compared to the MO controls at St32, when the miR‐181a/b morphant phenotype was first observed [Fig. 4(a)]. These data, together with the high conservation of the miR‐181 family seed sequence in the 3'UTR of the *Erk2* transcript [Fig. 4(b)], sustained the proposed miR181a/b targeting of these two genes (He et al., 2013; Wang et al., 2013). Consistent with these increased transcript levels, we detected a concomitant increase in the ERK2 protein levels in St32 miR‐181a/b morphant eyes [Fig. 4(c,d)].

The control of neurite outgrowth and elongation in neuronal cells by MAPK/ERK signaling involves a number of molecular events (Campbell and Holt, 2001; Campbell et al., 2001; Campbell and Holt, 2003; Jung et al., 2012). On specific stimuli, Mek1 kinase phosphorylates and activates ERK1/2 (Campbell and Holt, 2001). In turn, this activation produces a simultaneous increase in the phosphorylated forms of the eiF4E, eiF4E‐BP, and p70/S6K proteins, as well as an increase in the protein levels of cofilin/actin depolymerization factor (ADF) and RhoA, two well‐documented hallmarks of local actin organization that are necessary for axon specification and growth (Bradke and Dotti, 1999; Campbell and Holt, 2001; Campbell and Holt, 2003; Piper et al., 2006). We hypothesized that the MAPK/ERK signaling pathway is activated in miR‐181a/b morphants as a consequence of up‐regulation of the ERK2 protein. It has been reported that effective activation of MAPK/ERK signaling occurs upon up‐regulation of *Erk2* transcripts, which leads to a concomitant increase in both the total (ERK2) and activated phospho‐ERK2 (pERK2) forms, without changing their ratio (Schilling et al., 2009). In agreement with this hypothesis, Western blotting revealed that the levels of pERK2 were increased in miR‐181a/b morphants [Fig. 4(c–d)]. The latter led to a significant increase in the phosphorylated forms of eiF4E, eiF4E‐BP, and p70/S6K, as well as an increase in the ratio between the active‐phospho/ inactive‐total protein [Fig. 4(c–d)]. The levels of cofilin and RhoA, and the ratio between the active de‐phosphorylated and inactive phosphorylated forms of cofilin, were also significantly increased as a consequence of activation of MAPK/ERK signaling in miR‐181a/b morphant embryos [Fig. 4(c–e)]. Confirming the specificity of these findings we observed a decreased *Erk2* expression in miR‐181a/b overexpressing embryos (Supporting Information Fig. S5a). ERK2 is an important regulator of cell migration during gastrulation (Krens et al., 2008), and inhibition of ERK2 activity at the early stages of zebrafish development leads to dramatic embryogenesis defects, and the consequent embryonic lethality through prevention of blastula to gastrula transition (Krens et al., 2008). Consistent with the link between miR‐181a/b and ERK2, miR‐181a/b overexpression mostly phenocopied the defects reported for *Erk2* knock‐down [Supporting Information Fig. S5(b–d)], which resulted in lethality at gastrulation (78% ± 5% of injected embryos; *n* = 750). The relatively few surviving embryos (22% ± 5% of injected embryos; *n* = 750) showed a small body size and head defects, with enlargement of the otic vesicle, and in some cases, with complete absence of eye structures [Supporting Information Fig. S5(b,d)]. In agreement with these data, we found that in miR‐181a/b mimic‐injected embryos the active form of the MAPK‐cascade components were significantly down‐regulated compared to controls [Supporting Information Fig. S5(e–f)].

**Figure 5:**
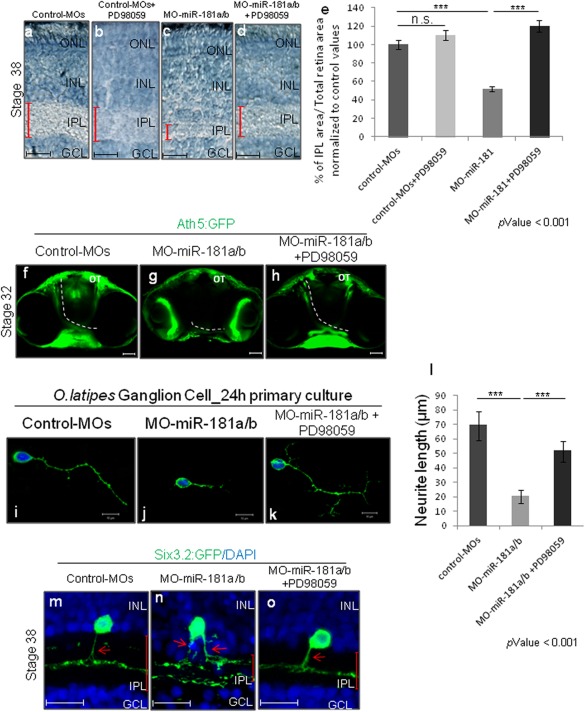
Down‐regulation of MAPK/ERK signaling activity rescue IPL, amacrine, and RGC neuritogenesis defects in miR‐181a/b‐morphants. **(a–d)** Retinal frontal sections of St38 untreated (a), PD98059‐treated (b) control‐MO embryos, and of untreated (c), PD98059‐treated (d) miR‐181a/b morphant embryos processed for Richardson‐Romeis staining. Red bars, IPL thickness. **(e)** Quantitative analysis of IPL thickness, defined as the mean ratio in the central retina between IPL and total retinal area normalized to IPL area on Total retina area ratio of Control‐MOs (*n* = 50). Data are means ± s.e.m. *** *p* < 0.001 (*t*‐tests). Note that addition of 25 μM PD98059 to miR‐181a/b morphants is sufficient to rescue the IPL morphant phenotype. **(f–h)** Representative 2‐D reconstruction of confocal images of St32 control‐MOs (f), miR‐181a/b morphant (g) and PD98059‐treated miR‐181a/b morphant (h) Ath5:eGFP transgenic whole‐heads. Dotted white lines mark optic nerve routes in control and miR‐181a/b morphants. Addition of PD98059 to miR‐181a/b morphants (h) was sufficient to rescue correct optic nerve growth in Ath5:eGFP morphant embryos. Scale bars: 50 µm. OT, optic tectum. **(i–k)** Representative images from primary RGC cultures from St30 control‐MOs (i), miR‐181a/b morphant (j), and PD98059‐treated miR‐181a/b morphant (k). The RGC axon length defect was rescued by treatment with PD98059 (k). Scale bars: 10 µm. **(l)** Quantification of RGC axonal length. Data are means + s.e.m (*n* = 100) from three independent cell culture experiments. ***p < 0.001 (*t*‐test). **(m–o)** Representative images of amacrine cells from St38 retinal sections of control‐MOs (m), miR‐181a/b morphant (n), and miR‐181a/b morphant treated with PD98059 Six3:eGFP transgenic embryos (o). Cell nuclei stained with DAPI (blue). GFP (green) stains amacrine cell soma and neurites. Red arrows, GFP‐labeled axon‐like structure of amacrine cells. Addition of PD98059 to miR‐181a/b morphants was sufficient to rescue neuritogenesis defects of miR‐181a/b morphant transgenic embryos. Red bars, IPL thickness. Scale bars: 20 µm. ONL, outer nuclear layer; INL, inner nuclear layer; GCL, ganglion cell layer. [Color figure can be viewed in the online issue, which is available at wileyonlinelibrary.com.]

These data suggest that miR‐181a/b‐mediated regulation of MAPK/ERK signaling controls local cytoskeletal rearrangements. If this is the case, inhibition of MAPK/ERK activity should counteract the impaired neuritogenesis observed in the miR‐181a/b morphant retinas. Treatment of st30 miR‐181a/b morphants with PD98059, a selective inhibitor of MAPK/ERK activation (Alessi et al., 1995), was sufficient to restore the levels of the active ERK2 protein and other MAPK/ERK components to values statistically indistinguishable from those of the control eyes [Fig. 4(c–d)]. As expected, an increase in the total ERK2 protein levels was still observed, as *Erk2* is a direct target of miR‐181a/b. This rescue of the MAPK/ERK cascade alteration was paralleled by the restoration of the ratio between the active dephosphorylated and inactive phosphorylated forms of cofilin [Fig. 4(e)]. Remarkably, exposure of miR‐181a/b morphant embryos to PD98059 rescued the retinal phenotype and the neuritogenesis defects (Fig. 5). Finally, administration of Y27632, a specific inhibitor of the RhoA‐ROCK pathway that links the MAPK/ERK pathway to local cytoskeleton rearrangements (Cheng et al., 2011), equally restored the miR‐181a/b morphant phenotype [Supporting Information Fig. S6(a–e)], compared to DMSO treatment. Control‐MOs treated with the same agents were morphologically indistinguishable from DMSO‐treated control‐MOs, supporting the specificity of this agent [Fig. 5(b), Supporting Information Fig. S6(b)]. Neither PD98059 nor Y27632 administration at st30 and onward significantly altered overall embryo growth and morphology, as compared with DMSO‐treated control‐MOs.

**Figure 6:**
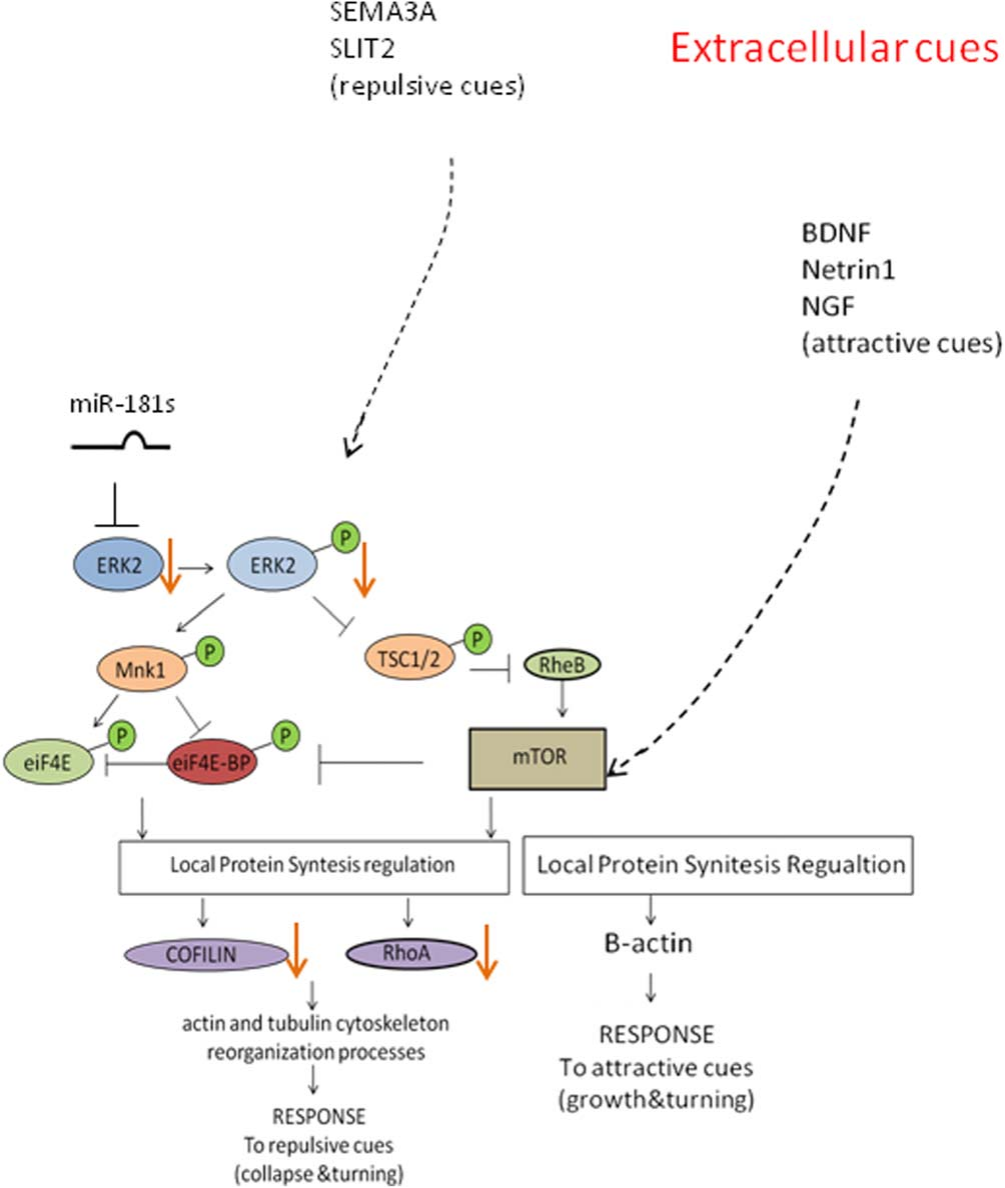
Model of miR‐181a/b function in retinal axon specification and growth. Diagram depicting miR‐181a/b activity on MAPK/ERK signaling. The miR‐181a/b‐mediated modulation of the MAPK signaling cascade leads to down‐regulation of the protein synthesis of RhoA and cofilin/ADF thus allowing neurite specification and rapid elongation. [Color figure can be viewed in the online issue, which is available at wileyonlinelibrary.com.]

Altogether, these data indicate that miR‐181a/b‐mediated regulation of MAPK/ERK signaling controls neuritogenesis in both amacrine cells and RGCs through local cytoskeletal rearrangement (Fig. 6).

## DISCUSSION

Regulation of axon specification and growth is critical for the correct wiring of the neural circuitry. However, the intracellular cascades that control the progressive assembly of these connections remain poorly understood. The use of a large variety of experimental approaches, such as morpholino injections, drugs treatment and *in vivo* and *ex vivo* strategies, allowed us to demonstrate the role of miR‐181a and miR‐181b in the control of retinal circuit assembly. In our study, we have demonstrated that miR‐181a and miR‐181b directly regulate amacrine cell axon specification and RGC axon growth through local cytoskeletal rearrangement, via targeting MAPK/ERK signaling. In support of this interpretation, pharmacological inhibition of MAPK/ERK and RhoA activities rescued neuritogenesis and the axon growth alterations observed in miR‐181a/b–depleted amacrine cells and RGCs.

The relevance of miR‐181a/b function in neuronal connectivity is also demonstrated by the alterations we observed in the OKR response assays in morphant larvae. MiR‐181a/b morphants showed alterations in optokinetic eye movements, with fewer complete optokinetic eye movement cycles. As the electrical activity in response to a light stimulus is not altered in the outer retina, demonstrated by the electroretinograms, this behavioral deficit is probably directly caused by defects in the visual system connectivity, such as alterations in either the IPL or the retino‐tectal projections, or in both of these structures.

Our findings show that miR‐181a/b are important for retinal axonal specification and growth, although we have not addressed whether they are also required for axon branching, dendrite polarization, or dendritic spine formation through a MAPK/ERK‐mediated, or indeed any other, mechanism. Pharmacological inhibition of MAPK/ERK activity increases neurite branching of dorsal root ganglion neurons (Jones et al., 2003). Interestingly, Neuropilin‐1 (Nrp1), which is a direct target of miR‐181b (Baudet et al., 2013), appears to bias the polarized extension of the dendrites of RGCs, through acting as a mediator of Semaphorin signaling during IPL formation (Kita et al., 2013). Of note, recent studies have shown that PTEN, which also contributes to axon growth and regeneration through the PTEN/mTOR and PTEN/Akt pathway (Park et al., 2008; Song et al., 2012), is also a miR‐181b–specific target (Henao‐Mejia et al., 2013), and that the miR‐181 family regulates the expression of MKP‐5, thereby facilitating p38MAPK phosphorylation (Song et al., 2013), which contributes to neurite outgrowth (Campbell and Holt, 2003). Similarly, the miR‐181 family appears to regulate Notch and Wnt signaling (Ji et al., 2009; Fragoso et al., 2012), which also control neuronal connectivity (Giniger, 2012; Park and Shen, 2012). Therefore, miR‐181a/b might have a key pleiotropic role in the control of the expression of components of different signaling pathways that are important in neural circuit formation.

miR‐181 family members have been previously reported to be regulated by TGF‐β at the transcription or processing level, depending on the cell type. In hepatocarcinogenesis, the TGF‐β effector SMAD4 appears to control the transcription of miR‐181b (Wang et al., 2010), whereas TGF‐β induces miR‐181a/b at the post‐transcriptional level through SMAD2/3‐dependent miRNA maturation in breast cancer (Wang et al., 2011). Interestingly, during early axon specification, *Tgfbr2* inactivation in mouse indicated a role for TGF‐β signaling in RhoA proteasomal degradation via the activation of the Par6/Smurf1 pathway (Yi et al., 2010). Based on the above observations, it is tempting to speculate that miR‐181a/b may represent a node between TGF‐ β and MAPK/ERK signaling, which were previously suggested to be linked during neurite specification and growth, but with controversial findings (Kim et al., 2004; Kerrison et al., 2005; Hocking et al., 2008; Walshe et al., 2011). However, further experiments are required to prove this hypothesis.

The conclusions of this study rely on data produced using multiple approaches, involving both morpholino‐based and nonmorpholino‐based strategies. The use of morpholinos for gene knockdown is very effective when dealing with genes/transcripts with multiple locations in teleost genomes, such as in the case of miR‐181a/b. Indeed, by morpholino we could rapidly and simultaneously inhibit all the miR‐181a/b medaka genomic copies in both wild‐type and in a number of transgenic medaka lines (Ath5:GFP, Six3:GFP, Six6:GFP), which allowed a detailed phenotypic analysis. The performance of all of the control experiments recommended when using morpholinos (Robu et al., 2007; Eisen and Smith, 2008) (see Supporting Text S1, Supporting Information Fig. S2), including the use of a mismatch‐MO as control in all the experiments, and the validation of our findings with the use of chemical drugs, were instrumental in consolidating our data and reinforce the notion that morpholinos are still valid tools to study gene function, as also recently reported (Stainier et al., 2015).

In conclusion, our findings provide a better understanding of the mechanisms underlying the wiring of the visual system, by unveiling a novel mechanism of miR‐181a/b‐mediated MAPK/ERK modulation in retina neurons. Our findings pave the way to future studies aimed at dissecting out the potential role of a miR‐181–regulated network in various neuronal cell type during CNS development.

## METHODS

### Medaka Fish Stocks

Ethics Statement: All studies on fish were conducted in strict accordance with the institutional guidelines for animal research and approved by the Italian Ministry of Health; Department of Public Health, Animal Health, Nutrition and Food Safety in accordance to the law on animal experimentation (article 7; D.L. 116/92; protocol number: 00001/11/IGB; approval date June 6, 2011). Furthermore, all animal treatments were reviewed and approved in advance by the Ethics Committee of the Institute of Genetics and Biophysics, IGB Animal House, (Naples, Italy).

Samples of the Cab strain of wild‐type medaka fish were kept and staged as described previously (Iwamatsu, 2004).

### Morpholinos and miR‐181 Mimic Injections

The MOs (Gene Tools, LLC) were designed and injected into fertilized one‐cell embryos, as detailed in Table S1. The specificity and inhibitory efficiency of each MO were determined as described previously (Eisen and Smith, 2008). Optimal MO concentrations (see Supporting Information Table S1) were determined on the basis of morphological criteria. miRIDIAN (Dharmacon) miRNA mimics for miR‐181 were injected at a final concentration of 2 μM. Embryos injected with mismatched MO‐miR‐181a/b or the negative mimic were used as controls.

### Whole‐Mount in‐situ Hybridization

Whole‐mount RNA *in‐situ* hybridization was performed, photographed, and sectioned as described previously (Conte and Bovolenta, 2007). The miRCURY detection miR‐181a/b Locked Nucleic Acid probes (Exiqon) were used according to Karali et al. (Karali et al., 2007).

### Richardson‐Romeis Staining (Histo‐Blue Sections)

The Richardson‐Romeis solution (1% Azur II solution, 1% methylene blue in 1% borax; 1:1) was applied briefly on slides on a heater (60°C). After the removal of the solution, the sections were washed briefly with tap water. The sections were left over night in water, dried on a heater, and closed with phosphate‐buffered saline (PBS)/ 50% glycerol.

### Immunofluorescence Analysis

For the immunofluorescence analysis on the medaka fish sections, the embryos were fixed overnight in 4% paraformaldehyde in PBS‐0.1%Tween (PTW) at 4°C, incubated overnight in 15% sucrose/ PTW at 4°C, and then incubated overnight in 30% sucrose/ PTW at 4°C. The cryosection of control and morphant medaka fish embryos were washed three times with PBS 1× (α‐Pax6, α‐Calretinin, α‐ Otx2) or PTW 1× (α‐Rhodopsin, α‐Syntaxin, α‐Zpr1, α‐GS6). Subsequently, the slides were boiled in citrate buffer (0.1 *M* citric acid, 0.1 *M* sodium citrate in water).

After an overnight incubation with the primary antibodies, the slides were incubated with the Alexa Fluor secondary antibodies (1:1000; Invitrogen). The slides were counterstained with 4,6‐diamidino‐2‐phenylindol (DAPI; Vector Laboratories). The immunofluorescence conditions for each antibody are reported in Supporting Information Table S2. The slides were photographed under LSM710 Zeiss confocal microscopy.

### Transgenic Lines

The Ath5:eGFP(Del Bene et al., 2007), Six3.2:eGFP (Conte and Bovolenta, 2007), and Six6:eGFP transgenic lines were used to analyze the amacrine cells and RGCs. The Six6:eGFP line was obtained using the enhancer of Six6 (Conte et al., 2010b) to drive the expression of a cytoplasmic eGFP in all of the amacrine cells. Transgenic embryos were injected with mismatched MO‐miR‐181a/b (control MO) and MO‐miR‐181a/b (morphants). The embryos were then fixed at the stages of interest by an overnight incubation in 4% paraformaldehyde in PTW at 4°C, and then incubated overnight in 15% sucrose/PTW at 4°C, and then again incubated overnight in 30% sucrose/PTW at 4°C. Cryosections of the control and morphant transgenic embryos were washed three times with PTW and were counterstained with DAPI (Vector Laboratories). The slides were photographed under LSM710 Zeiss confocal microscopy.

### Real‐Time PCR

Total RNA from St32 eyes were obtained from control, miR‐181a/b morphant, PD98059‐treated miR‐181a/b morphant embryos. For negative mimic and mimic‐181 overexpressing analysis, the total RNAs were extracted from whole embryos. The RNAs were extracted and digested with DNaseI using RNeasy extraction kits, according to the manufacturer instructions. The cDNAs were generated using Quantitect kits for qRT‐PCR analysis. The qRT‐PCR reactions were performed with nested primers and carried out with the Roche Light Cycler 480 system. The quantification data are expressed in terms of the cycle threshold (Ct). The Ct values were averaged for each triplicate. The *olHprt* and *olGapdh* genes were used as the endogenous controls for the experiments. The *olProx1* gene was used as the positive control, as it is an already validated target for miR‐181 (Kazenwadel et al., 2010), while *olWnt11* was used as the negative control. The primer sequences are reported in Table S1.

### Protein Isolation and Western Blotting

The embryos were dechorionated and deyolked. Total protein extract from St32 eyes were obtained from control, miR‐181a/b morphant, miR‐181a/b morphant treated withPD98059. For negative mimic and mimic‐181 overexpressing analysis, the total protein were extracted from whole embryos The proteins were extracted in RIPA buffer (50 mM Tris‐HCl, 1 mM EDTA, 150 mM NaCl, 1% Triton‐100X, 0.1% SDS, protease inhibitor cocktail tablet [Roche]). The protein extract concentrations were determined using the Bio‐Rad protein assay (Bio‐Rad, Munich, Germany). A total of 20–40 µg protein from each sample was loaded on 12% or 15% SDS‐polyacrylamide gels. For western blotting, the gels were electroblotted onto nitrocellulose filters and sequentially immunostained with the primary antibodies overnight at 4°C, reported in Supporting Information Table S3, and then with peroxidase‐labelled secondary antibodies (GE Healthcare, Little Chalfont Buckinghamshire, UK), at room temperature for 1–2 h. The Western blotting was revealed using the Pierce ECL Western blotting substrate (Thermo Scientific), and the images were acquired using the Chemidoc‐IT UVP and the Visionworks Software. The Western blotting data were quantified using the ImageJ analysis package (National Institutes for Health). The signals for each protein staining were quantified and then normalized for the GAPDH in the same sample (internal normalization). These normalized values were then compared to the values in the control sample. The average of the normalized values from three different experiments is illustrated as the relative fold change.

### Primary Culture of Medaka Fish Retinal Cells

For the generation of *in‐vitro* primary cultures of amacrine cells, we used the Six6:eGFP transgenic medaka line, in which the GFP is expressed in all of the amacrine cells, thus allowing the visualization of a greater number of amacrine cells, with respect to the use of the Six3:eGFP line. Eyes extracted from medaka Six6:eGFP control and morphant embryos at St32 (around the onset of amacrine cell differentiation) were dissociated in 100 µL L15 medium supplemented with 10% fetal bovine serum, 100 U/ml penicillin and 50 mg/mL streptomycin, with 20 µL 10 mg/mL Trypsin (in PBS), and incubated at 37°C (shaken periodically). After the addition of 20 µL soya bean trypsin inhibitor (20 mg/mL in PBS), mechanical dissociation was obtained using a syringe with a G27 needle. The cells were seeded onto 13 mm coverslip‐bottomed dishes covered with 20 µg/mL poly‐D‐lysine (bidistilled water) and 10 µg/mL laminin (in PBS), in 600 µL complete L15 + 20 µL N2 supplement medium (100×), preheated at 37°C. The cells were then kept at 30°C for 24 h. The same protocol was used for the dissociation of Ath5:eGFP control and morphant eyes at St30 (around the onset of RGC differentiation).

### Drug Treatments

The chorion was removed with the hatching enzyme. St30 morphant or control embryos were grown in 25 µM PD98059, or 100 nM Y27632 diluted in 3%DMSO, 1×Yamamoto, for 24 h or 6‐days. For the control experiments, the St30 morphant or control embryos were grown in 1×Yamamoto/3%DMSO.

### Optokinetic Response

Medaka fish larvae were immobilized dorsal‐up in the center of 35‐mm‐diameter Petri dishes containing prewarmed (28°C) 3% methylcellulose, to constrain whole‐body movement without significantly affecting eye movement. The OKR was evoked in a similar way to that described previously (Rinner et al., 2005; Huber‐Reggi et al., 2013). Using an LCD projector (PLV‐Z3000; Sanyo), a computer‐generated visual stimulus was projected via a wide‐angle conversion lens and a mirror to the internal walls of a paper drum (diameter, 9 cm) mounted on a transparent glass plate. The embedded larva was placed in the center of the drum and was illuminated from below with infrared‐emitting diodes (*λ*_peak_ = 940 nm, BL0106–15–28; Kingbright). The OKR was elicited with a computer‐generated (Straw, 2008) black and white sine‐wave grating pattern with 85% contrast (maximum illumination, 400 lux), a spatial frequency of 20 cycles/ 360°, and an angular velocity of 7.5°/s. The stimulation lasted for 180 s, whereby the direction of the moving grating changed every 60 s. During the visual stimulation, the eye movements were recorded using an infrared‐sensitive CCD camera (Guppy F‐038B NIR, Allied Vision Technologies), and tracked based on pixel intensity by custom‐developed software based on Lab‐View 2009 and NI Vision Development Module 2009 (National Instruments). The frames were processed simultaneously, with a frame rate of 25 frames/s.

### Electroretinograms

Electroretinograms were recorded as described previously (Makhankov et al., 2004). Briefly, larvae were dark adapted for 30 min. For recording, a reference electrode was placed in 1% agarose in dd H_2_O. The larva was placed dorsal‐up on moist paper covering the reference electrode. The recording electrode with a tip diameter of 20 μm was filled with E3 and placed on the cornea of the larva. Light stimuli of 100 ms with interstimulus intervals of 7 s were applied. The light stimulus intensity was 665 lux.

## Supporting information

Supplementary Information

Supplementary Information

Supplementary Information
