# Supplementary material for: miR‐181a/b control the assembly of visual circuitry by regulating retinal axon specification and growth
Source: Dev Neurobiol. 2015 Jun 11;75(11):1252–67. doi: 10.1002/dneu.22282 (PMC5033011; doi:10.1002/dneu.22282)
Supplement: Supplementary file 3 — Supplementary Information [file DNEU-75-1252-s003.pdf]

Supporting Text S1

Controls for MO injections

MO-miR-181a and the MO-miR-181b were designed complementary to the miR-181a and miR-181b mature sequences, respectively. The control-MOs were two MOs with the substitutions of five bases (indicated in red in Table S1), mismatched (mm)-MO-miR-181a with respect to MO-miR-181a, and mismatched (mm)-MO-miR-181b with respect to MO-miR-181b. We used 5 nucleotide mismatch MOs as controls in the experiments because these are most similar to the experimental MOs. Of note, we did not observe any qualitative and quantitative phenotypic effects following injection of mm-MO-miR-181a or mm-MO-miR-181b; hence, the subsequent studies were performed using the mismatched MOs as control. The blocking efficiencies of MO-miR-181a and MO-miR-181b were verified using a 'direct assay', based on the ability of the MO to interfere with the translation of a reporter, in which the 5'UTR of the EGFP coding sequence was fused to the portion of ol-miR-181a and ol-miR-181b that showed complete sequence complementarity with respect to the two designed MOs (Figure S2). Injections of both the MO-miR-181a and MO-miR-181b led to the specific absence of EGFP signal of the corresponding reporter construct (Figure S2 c-d') compared to the EGFP signals of the constructs injected alone (Figure S2 a-b') or co-injected with mm-MO-miR-181a or mm-MO-miR-181b (Figure S2 e-f'). Moreover, we also verified that injections of MO-miR-181a did not lead to repression of the EGFP signal of the miR-181b reporter construct (Figure S2 g-g'). The absence of EGFP signal repression was also observable when MO-miR-181b was injected with the miR-181a reporter construct (Figure S2 h-h'), which demonstrated that each MO specifically recognised its targets. Analysis of the inhibitory efficiency of each MO was performed by quantifying EGFP intensity, as reported previously[1], and with ImageJ analysis (National Institutes of Health). As injected MOs bind to mature miRNA sequences, which makes them inaccessible to the locked nucleic acid probes, in *in-situ* hybridization[2], the efficiency of MO inhibition on endogenous miRNA activity was assessed by *in-situ* hybridization. This analysis showed that the mature miRNA signals was completely suppressed after injection of the MOs (Figure S2 i-l), which strongly indicated a high knock-down efficiency for both of the MOs in these experiments.

Activation of p53 is an occasional off-targeting effect of knock-down strategies that induce neuronal p53-dependent cell death accompanied by abnormal tissue development, such as microcephaly, microphthalmia, and others[3]. In this case, the phenotype can be rescued by co-injection of a MO against p53[4]. Although morphant embryos did not show any morphological

differences compared to both wild-type and control (mm-MO-miR-181a/b) embryos, we further excluded that the ocular phenotype in morphants was not related to non-specific neural cell death in response to the injection of our MO. Therefore, we co-injected MO-miR-181a and MO-miR181b with an MO against p53 (MO-p53)[5]. Of note, there were no modifications of the morphant ocular phenotype, which further confirmed the specificity of the MO-miR-181a/b targeting and the absence of the occasional off-targeting effect due to the knock-down strategy.

1. Esteve, P. . *et al.* SFRP1 is required for the proper establishment of the eye field in the medaka fish. *Mechanisms of Development* 121, 687-701, doi:10.1016/j.mod.2004.03.003 (2004).
2. Kloosterman WP *et al.* Targeted inhibition of miRNA maturation with morpholinos reveals a role for miR-375 in pancreatic islet development. *PLoS Biol.* 2007 Aug;5(8):e203.
3. Robu, M. E. *et al.* p53 activation by knockdown technologies. *PLoS Genetics* 3, e78, doi:10.1371/journal.pgen.0030078 (2007).
4. Eisen, J. S. & Smith, J. C. Controlling morpholino experiments: don't stop making antisense. *Development* 135, 1735-1743, doi:10.1242/dev.001115 (2008).
5. Conte, I. *et al.* miR-204 is required for lens and retinal development via Meis2 targeting. *Proc Natl Acad Sci U S A* 107, 15491-15496, doi:0914785107 [pii].

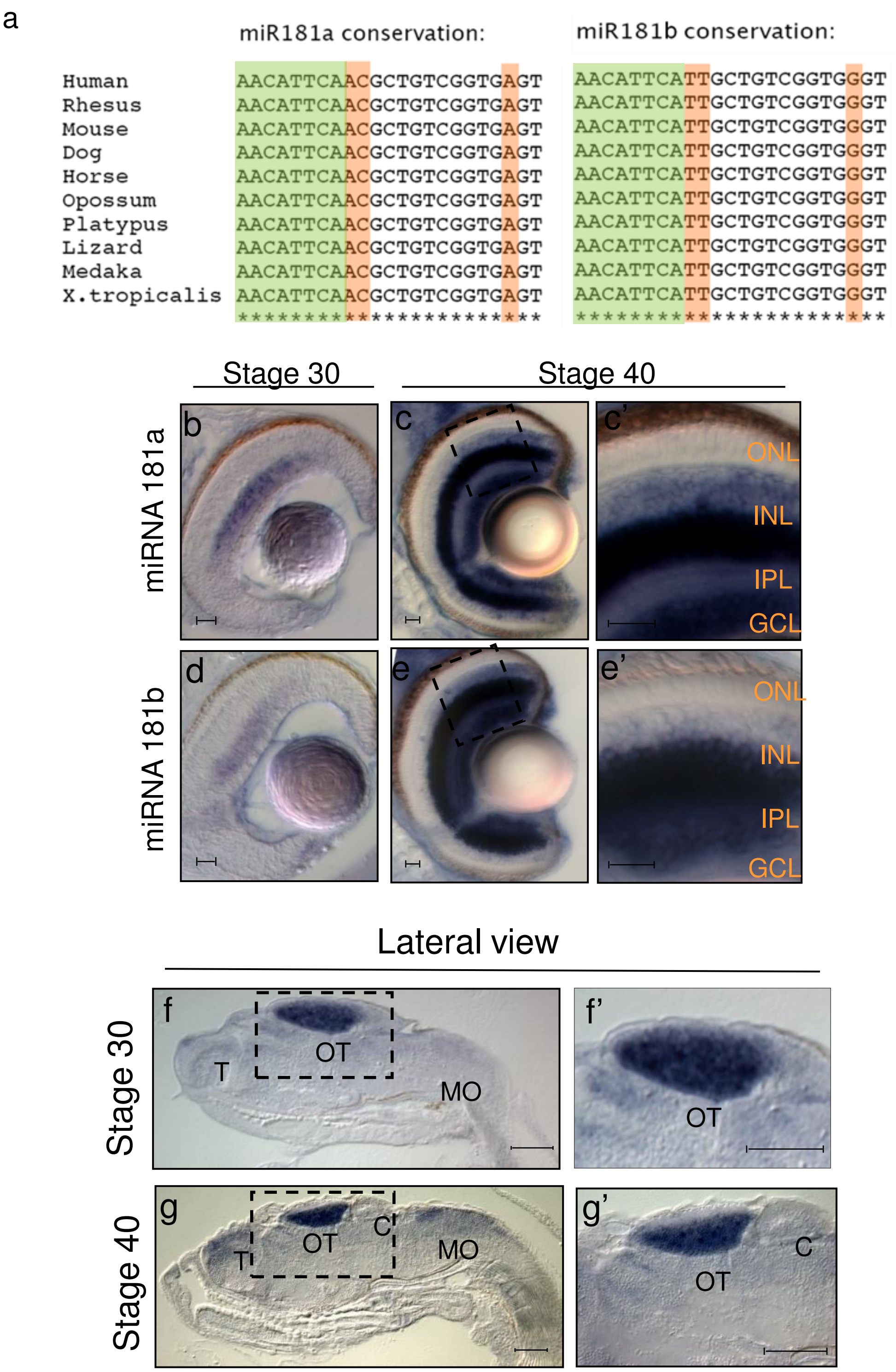
a
miR181a conservation:
miR181b conservation:
Human
Rhesus
Mouse
Dog
Horse
Opossum
Platypus
Lizard
Medaka
X.tropicalis
AACATTCAACGCTGTCGGTGAGT
AACATTCATTGCTGTCGGTGGGT
Stage 30
Stage 40
miRNA 181a
miRNA 181b
b
c
c'
d
e
e'
ONL
INL
IPL
GCL
Lateral view
Stage 30
Stage 40
f
f'
g
g'
T
OT
MO
C

Figure S1

Figure S1. Expression profiling of miR-181a and miR-181b in the developing retina and CNS. **(a)** Alignment of mature miR-181a and miR-181b sequences between different species. Note that miR-181a and miR-181b differ by three bases (highlighted in orange), which are located outside the seed region (highlighted in green). **(b-e')** RNA *in-situ* hybridization for miR-181a (b-c') and miR-181b (d-e') at St30 (b, d) and St38 (c, c', e, e'). At St30, expression is detected for the differentiating amacrine and ganglion cells of the neural retina. At St38, miR-181a and miR-181b are expressed in the inner nuclear layer (INL) and ganglion cell layer (GCL), with the strongest detection signal where amacrine cells are located. (c') and (e') are higher magnifications of the boxed areas in (c) and (e), respectively. ONL, outer nuclear layer; IPL, inner plexiform layer. Scale bars: 20 µm. **(f-g')** Lateral sections of miR-181 RNA *in-situ* hybridization at St30 and St38, showing the distribution of their expression in the central nervous system, with the highest levels in the optic tectum (OT). T, telencephalon; MO, medulla oblongata. Scale bars: 50 µm.

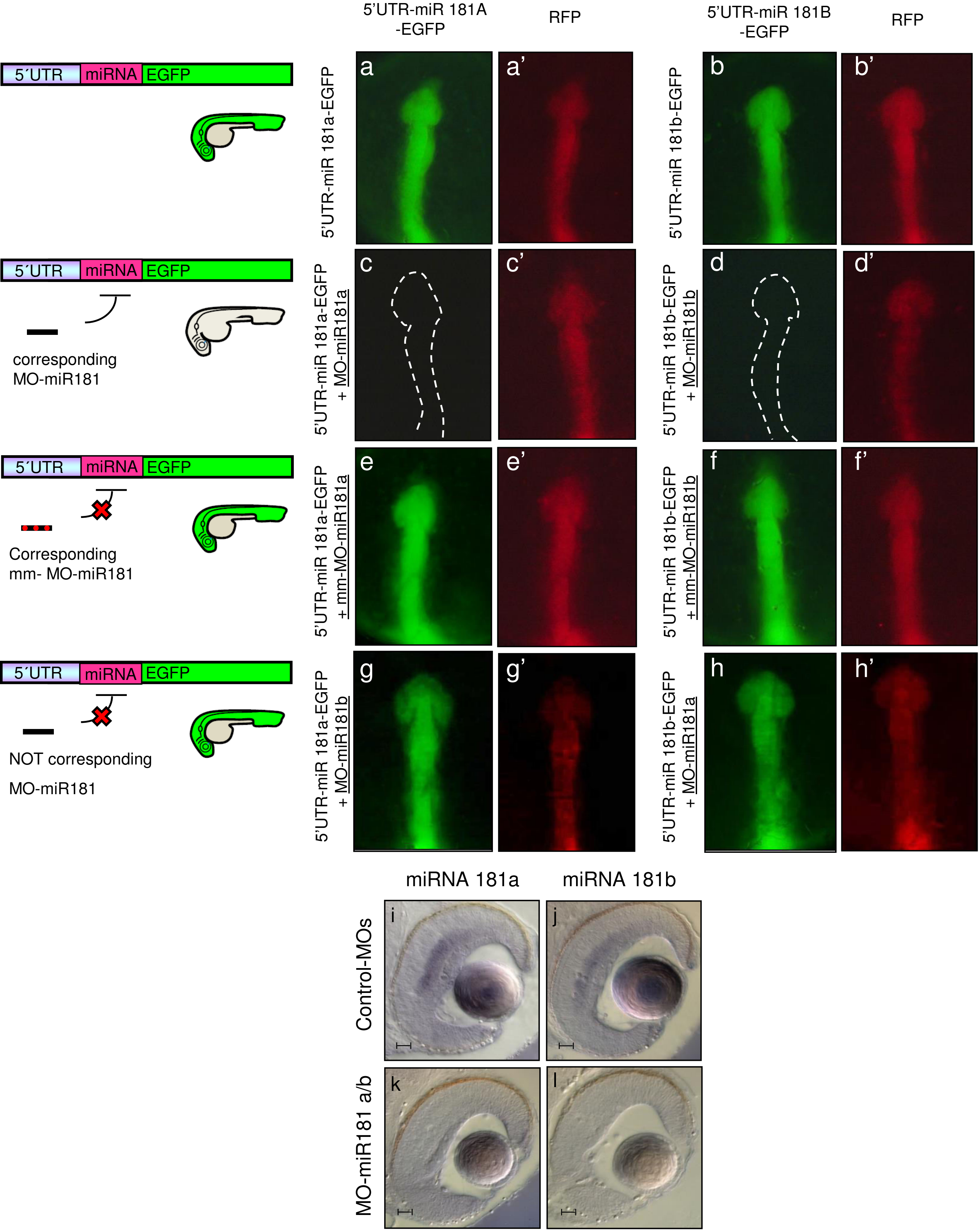
5'UTR-miR 181A -EGFP
RFP
5'UTR-miR 181B -EGFP
RFP
5´UTR
miRNA
EGFP
corresponding MO-miR181
Corresponding mm- MO-miR181
NOT corresponding MO-miR181
5'UTR-miR 181a-EGFP
5'UTR-miR 181b-EGFP
5'UTR-miR 181a-EGFP + MO-miR181a
5'UTR-miR 181b-EGFP + MO-miR181b
5'UTR-miR 181a-EGFP + mm-MO-miR181a
5'UTR-miR 181b-EGFP + mm-MO-miR181b
5'UTR-miR 181a-EGFP + MO-miR181b
5'UTR-miR 181b-EGFP + MO-miR181a
miRNA 181a
miRNA 181b
Control-MOs
MO-miR181 a/b

Figure S2

Figure S2. Evaluation of morpholino efficacy and specificity.

Left: Schematic representations of the direct assay used to assess the efficiency of the MO-miR-181a and MO-miR-181b, which are designed to block the miR-181a and miR-181b mature forms, respectively (see Supporting Text S1 for more details). **(a-h')** Representative medaka embryos injected with the synthetic mRNAs encoding the 5'UTR-miR-181a-EGFP or the 5'UTR-miR-181b-EGFP reporter construct alone (a, b) or together with MO-miR-181a and MO-miR-181b, respectively (c, d). Note that the 0.03 mM concentration of MO-miR-181a or MO-miR-181b was sufficient to completely abolish EGFP reporter expression. This effect is highly specific, because co-injections of the reporter construct with the mismatch mm-MO-miR-181a or mm-MO-miR-181b (e, f) or with the non-corresponding MO (g, h) did not decrease the EGFP expression. Red fluorescent protein serves as the injection control. **(i-l)** Frontal vibratome sections from St30 control-MOs (i, j) and MO-miR-181a/b–injected (k, l) embryos hybridized with digoxigenin-labeled miR-181a (i, k) and miR-181b (j, l) specific probes. MiR-181a and miR-181b expression were not detected in morphant embryos. Scale bars: 20 µm.

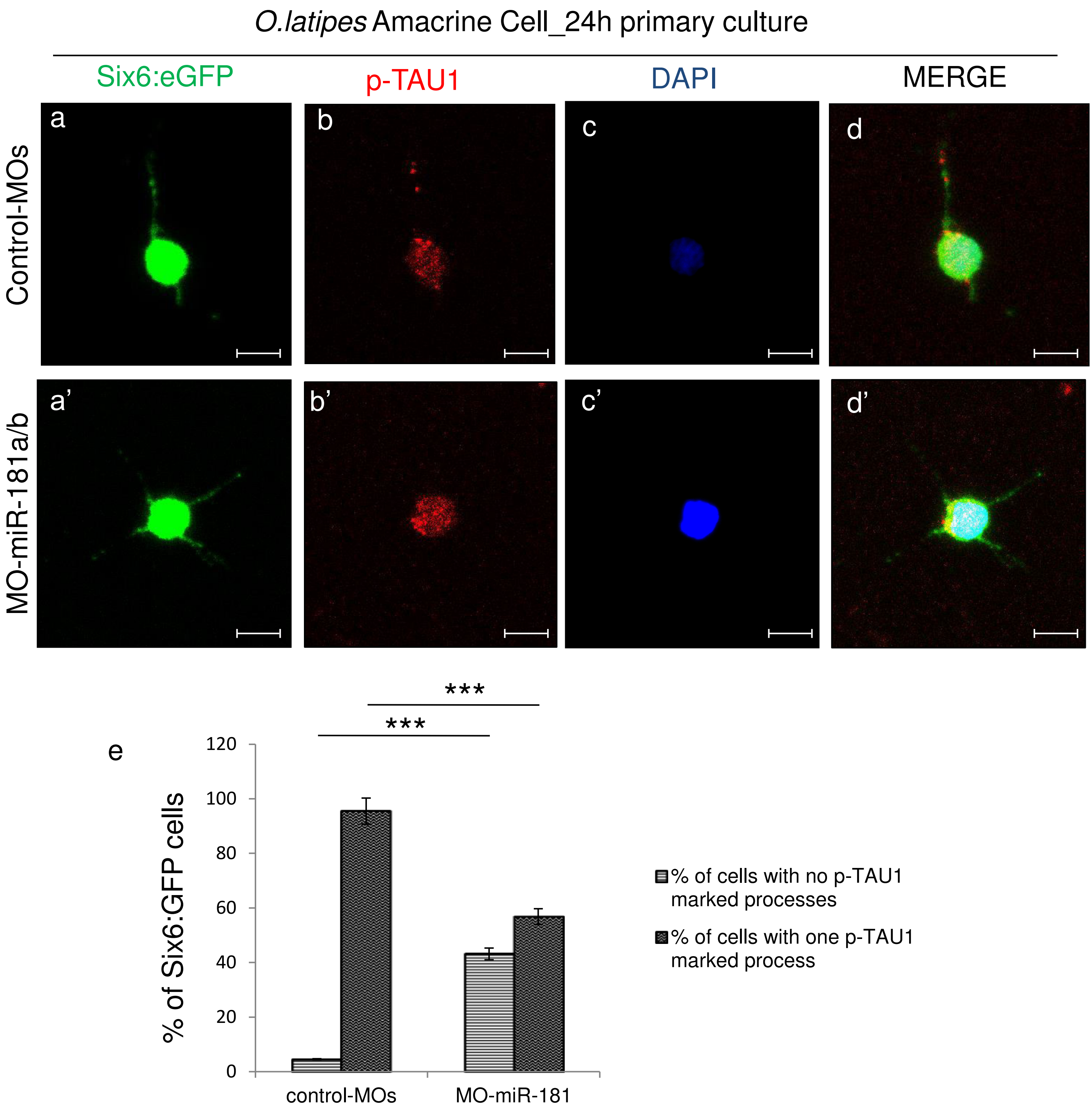

Figure S3. MO-miR-181a/b amacrine cells do not specify axon-like structures.
(a-d’) Twenty-four-hour primary cultures of amacrine cells dissected from St32 control-MOs (a-d) and MO-miR-181a/b Six6:GFP transgenic retinas (a’-d’). Staining with the anti-phospho-TAU1 antibody (b, b’, red) allows to visualize their axon-like structures. In control cells (a-d), only one process is stained by phospho-TAU1 (b), whereas in the morphant amacrine cells there are multiple processes emerging from the single cell body, none of which is stained for phospho-TAU1 (b’). Scale bars: 10 μm. **(e)** Quantification of the number of axon-like processes stained for phospho-TAU1, as in (a-d’). Data are means ±SEM (n = 100, for each). *** $p < 0.0001$, (likelihood ratio test for Negative Binomial generalized linear models).

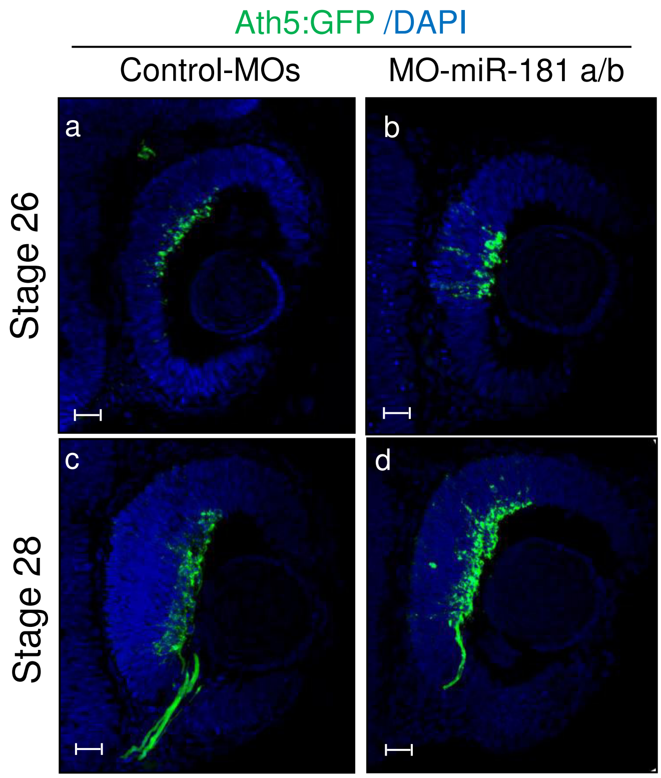

Figure S4. Down-regulation of miR-181a or miR-181b results in a partial decrease in optic nerve length, which is not due to alterations in RGC differentiation.
(a-c) Frontal sections of St26 and St28 control-MOs (a, c) and miR-181a/b morphant (b, d) Ath5:eGFP transgenic medaka retinas. Cell nuclei are stained with DAPI (blue). GFP (green) labels RGC soma and neurites. No differences were observed between control and morphant embryos, which indicates that miR-181a/b down-regulation does not alter retinal ganglion cell differentiation. Scale bars: 20 µm.

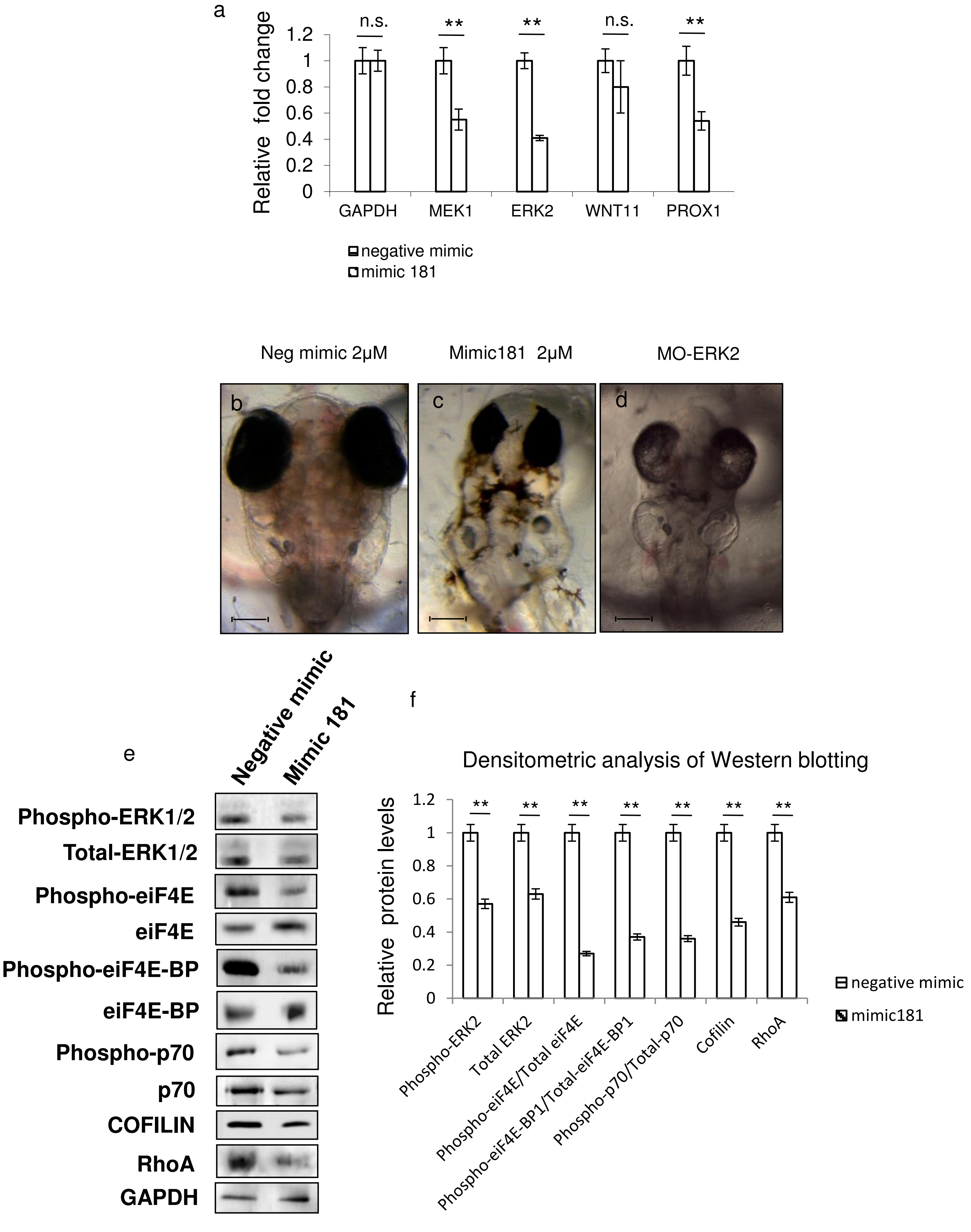
a
n.s.
**
**
n.s.
**
Relative fold change
1.2
1
0.8
0.6
0.4
0.2
0
GAPDH
MEK1
ERK2
WNT11
PROX1
negative mimic
mimic 181
Neg mimic 2μM
Mimic181 2μM
MO-ERK2
b
c
d
Negative mimic
Mimic 181
e
Phospho-ERK1/2
Total-ERK1/2
Phospho-eiF4E
eiF4E
Phospho-eiF4E-BP
eiF4E-BP
Phospho-p70
p70
COFILIN
RhoA
GAPDH
f
Densitometric analysis of Western blotting
Relative protein levels
Phospho-ERK2
Total ERK2
Phospho-eiF4E/Total eiF4E
Phospho-eiF4E-BP1/Total-eiF4E-BP1
Phospho-p70/Total-p70
Cofilin
RhoA
negative mimic
mimic181

Figure S5

Figure S5. MiR-181 over-expression results in alterations of early embryo development and leads to strong down-regulation of the MAPK/ERK cascade.

(a) qRT-PCR on total RNA for Erk2, Wnt11 and Prox1 transcripts from St16 control (negative mimic) and mimic miR-181-injected embryos, normalized to GAPDH transcript levels. Erk2 levels were decreased in miR-181 over-expressing embryos. Prox1, an already validated miR-181 target, was the positive control, and Wnt11 as the negative control. Data are means ± SEM. **, p <0.01. **(b-d)** Representative images of Medaka miR-181a/b over-expressing embryos (c), with the control embryos injected with a negative mimic (b). The miR-181a/b over-expressing embryos (mimic181) showed a prominent and marked phenotype characterized by lethality at gastrulation in the majority of cases. The few surviving embryos showed small body size, head defects with enlargement of otic vesicles, and in some cases, complete absence of eye structures (b), which parallels the phenotype observed in *Erk2* morphant embryos[30] (d). **(e, f)** Western blotting (e) and quantification by densitometric analysis (f) shows that at St16 the total ERK2 and the phospho-ERK2 levels are decreased in the presence of the miR-181a/b mimic, with respect to negative-mimic–injected embryos. This results in a decrease of downstream effectors activation, including the phosphorylated forms of eiF4E, eiF4E-BP, and p70/S6K, finally leading to a reduction in cofilin and RhoA levels. Data are means ±/SEM **, p <0.01.

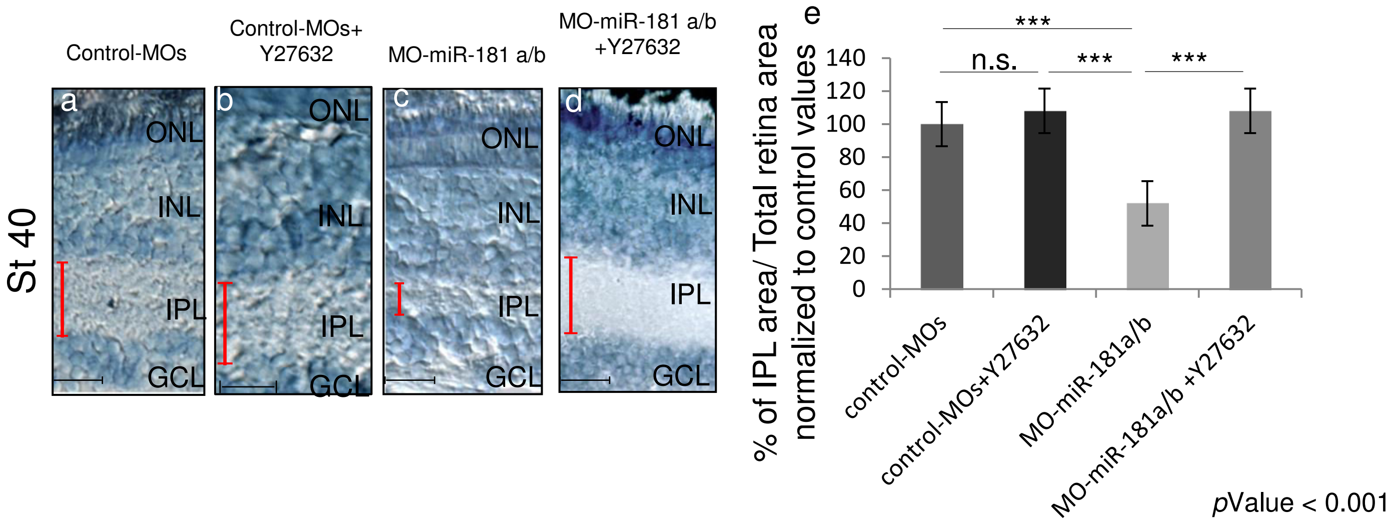

Figure S6. The increased activity of RhoA-ROCK pathway plays a role in the MO-miR-181a/b retinal phenotype.

(a-e) Representative retinal frontal sections of St38 control-MOs (a), Y27632-treated control-MOs (b), miR-181a/b morphant (c), Y27632-treated miR-181a/b morphant (d) embryos processed for Richardson Romeis staining. Red bars, thickness of the inner plexiform layer (IPL). Y27632, an inhibitor of RhoA activity, rescues retinal IPL thickness defects in MO-miR-181a/b embryos, indicating that increased activity of RhoA has a role in the MO-miR-181a/b phenotype. Scale bars: 20 µm. ONL, outer nuclear layer; INL, inner nuclear layer; GCL, ganglion cell layer. **(e)** Quantitative analysis of IPL thickness, as the ratio in the central retina between the IPL area and Total retinal area normalized to IPL area on Total retina area ratio of Control-MOs. Data are means ±s.e.m. ***, P <0.001 (t-tests).

Supporting Movie Legends

Movie S1. Representative movie of 30 s of the optokinetic response test in control-MOs larvae. Figure 3a summarizes the results of the entire test.

Movie S2. Representative movie of 30 s of the optokinetic response test in miR-181a/b morphant larvae. Note the alteration in the saccadic movements. Figure 3a summarizes the results of the entire test.

Table S1: Sequences of used Morpholinos (MOs) and Oligonucleotides primers

MOs	Sequence	Concentration (mM)
MO-miR-181a	5′-AACTCACCGACAGCGTTGAATGTTC-3’	0.12
MO-miR-181b	5′-AACCCACCGACAGCAATGAATGTTG-3’	0.12
mm-MO-miR-181a	5′-AAGTCAGCGACACCGTTCAATCTTC-3’	0.12
mm-MO-miR-181b	5′-AAGCCACGGACACCAATCAATCTTG-3’	0.12
MO-olp53	5′-CGGGAATCGCACCGACAACAATACG-3’	0.09
MO-ERK2	5′-CTCAGTCTAAATGGCGTTACCTTCA-3’	0.4
**Oligonucleotides primers**		
Ol miR181a/Fc	5’-GATCGAACATTCAACGCTGTCGGTGAGTT-3’	
Ol miR181a/Rc	5’- TAGCAACTCACCGACAGCGTTGAATGTTC -3’	
Ol miR181b/Fc	5’-GATCAACATTCATTGCTGTCGGTGGGTT -3’	
Ol miR181b/Rc	5’-TAGCAACCCACCGACAGCAATGAATGTT -3’	
Ol HPRTrt-F	5'- CTGAACAGGAACAGCGACC-3'	
Ol HPRTrt-R	5'- TGAGGAGCTCCAATAACGTC-3'	
Ol GAPDHrt-F	5'- CGGCAAGCTGATAGTCGATG-3'	
Ol GAPDHrt-R	5'- AGAAACACTCCGGTGGACTC-3'	
Ol ERK2rt-F	5'- GCAGCGACAGCAGATAGTTC -3'	
Ol ERK2rt-R	5'- GCCGAGATGTTGTCCAACAG -3'	
Ol PROX1rt-F	5'- ATCTCACCTTACTCAGGCAG-3'	
Ol PROX1rt-R	5'- TAAGCATGTTGGAGCTTGGG-3'	
Ol WNT11rt-F	5'- CCGATGCTCCCATGAAGATG -3'	
Ol WNT11rt-R	5'- CAGGATCCAGATACACCATG -3'	
Ol pri-miR-181a/chr4rt-F	5'-GAGTGAATATGAGGGGTTAG -3'	
Ol pri-miR-181a/chr4rt-R	5'-CAAGAACAGAGCTGATGGTG-3'	
Ol pri-miR-181b/chr4rt-F	5'- GCTGCAGTGTGTTGATGGAG -3'	
Ol pri-miR-181b/chr4rt-R	5'- GTGAGTTTATACCAACCATG-3'	
Ol pri-miR-181a/chr17rt-F	5'-CATGCTTGTGTTGCACAACC -3'	
Ol pri-miR-181a/chr17rt-R	5'-GCTGCGACCCTGAGCTGAGC -3'	
Ol pri-miR-181b/chr17rt-F	5'-CTGCCTGACTTGTGTCTCAG -3'	
Ol pri-miR-181b/chr17rt-R	5'-GAGAGGGGAAAATAGAAGTG -3'	
Ol pri-miR-181a/chr9rt-F	5'- CAATGAATGTCCGAATGTCTC-3'	
Ol pri-miR-181a/chr9rt-R	5'- GCGGAATCCAGTTTTTGTAG -3'	
Ol pri-miR-181b/chr9rt-F	5'- GCAAGCTGCTCTATGAAATG -3'	
Ol pri-miR-181b/chr9rt-R	5'- CCTGATAGGGGTTGACCAAC -3'	

Ol pri-miR-181a/U105rt-F	5'- CAAATGAGGCATCAACCACC -3'
Ol pri-miR-181a/U105rt-R	5'- GACTAGAGGATGGGGCGGTC -3'
Ol pri-miR-181b/U105rt -F	5'- TGTGAATTTACTGACCTTTG -3'
Ol pri-miR-181b/U105rt-R	5'- ACTCGCCAAGAAATATTAAG -3'

Table S2: Immunofluorescence conditions used for each antibody

Antibody	Supplier	Boiling (min)	Blocking buffer	Dilution solution	Dilution	Secondary antibody
Rhodopsin	Abcam	1	10% FBS/ PTW (1x)	5% FBS/ PTW (1x)	1:5000	α-mouse
Zpr1	Zfin	2	5% goat serum/ 0.5% Triton in PBS (1x)	5% FBS/ PTW (1x)	1:200	α-mouse
Syntaxin	Sigma	2	10% FBS/ PTW (1x)	5% FBS/ PTW (1x)	1:100	α-mouse
Otx2	Abcam, ab21990	10	10% FBS/ PTW (1x)	5% FBS/ PTW (1x)	1:100	α-rabbit
GS6	Millipore	2	10% FBS/ PTW (1x)	5% FBS/ PTW (1x)	1:100	α-mouse
Pax6	Covance, Princeton, NJ	8	10% FBS/ PTW (1x)	5% FBS/ PTW (1x)	1:250	α-rabbit
Calretinin	SWANT (6B3)	5	5% FBS/ 0.3% Triton/ PBS (1x)	1% BSA/ 0.3% Triton/ PBS (1x)	1:500	α-mouse
ERK1/2	Cell signaling	5	5% FBS/ 0.3% Triton/ PBS (1x)	1% BSA/ 0.3% Triton/ PBS (1x)	1:50	α-rabbit

FBS, fetal bovine serum

PTW, PBS/0.1%Tween

BSA, bovine serum albumin

Table S3: Western blotting conditions used for each antibody

Antibody	Dilution	Supplier
Total ERK1/2	1:1000	Cell Signaling
Phospho-ERK1/2	1:1000	Cell Signaling
Total eiF4E	1:500	Cell Signaling
Phospho-eiF4E	1:500	Cell Signaling
Total eiF4E-BP1	1:500	Cell Signaling
Phospho-eiF4E-BP1	1:500	Cell Signaling
Total p70	1:1000	Cell Signaling
Phospho-p70	1:100	Cell Signaling
Cofilin	1:500	Cell Signaling
Phospho-cofilin	1:500	Cell Signaling
RhoA	1:200	Millipore
GAPDH	1:500	Santa Cruz
